# A review of genetic resources and trends of omics applications in donkey research: focus on China

**DOI:** 10.3389/fvets.2024.1366128

**Published:** 2024-10-11

**Authors:** Muhammad Zahoor Khan, Wenting Chen, Xinrui Wang, Huili Liang, Lin Wei, Bingjian Huang, Xiyan Kou, Xiaotong Liu, Zhenwei Zhang, Wenqiong Chai, Adnan Khan, Yongdong Peng, Changfa Wang

**Affiliations:** ^1^Liaocheng Research Institute of Donkey High-Efficiency Breeding and Ecological Feeding, Liaocheng University, Liaocheng, China; ^2^Genome Analysis Laboratory of the Ministry of Agriculture, Agricultural Genomics Institute at Shenzhen, Chinese Academy of Agricultural Sciences, Shenzhen, China

**Keywords:** omics application, donkey breeds, genetic resources, production traits, reproductive traits, microbiota, molecular breeding

## Abstract

Omics methodologies, such as genomics, transcriptomics, proteomics, metabolomics, lipidomics and microbiomics, have revolutionized biological research by allowing comprehensive molecular analysis in livestock animals. However, despite being widely used in various animal species, research on donkeys has been notably scarce. China, renowned for its rich history in donkey husbandry, plays a pivotal role in their conservation and utilization. China boasts 24 distinct donkey breeds, necessitating conservation efforts, especially for smaller breeds facing extinction threats. So far, omics approaches have been employed in studies of donkey milk and meat, shedding light on their composition and quality. Similarly, omics methods have been utilized to explore the molecular basis associated with donkey growth, meat production, and quality traits. Omics analysis has also unraveled the critical role of donkey microbiota in health and nutrition, with gut microbiome studies revealing associations with factors such as pregnancy, age, transportation stress, and altitude. Furthermore, omics applications have addressed donkey health issues, including infectious diseases and reproductive problems. In addition, these applications have also provided insights into the improvement of donkey reproductive efficiency research. In conclusion, omics methodologies are essential for advancing knowledge about donkeys, their genetic diversity, and their applications across various domains. However, omics research in donkeys is still in its infancy, and there is a need for continued research to enhance donkey breeding, production, and welfare in China and beyond.

## 1 Introduction

In the field of biology, the term “omics” is commonly used to denote scientific disciplines focused on the comprehensive characterization of molecular components originating from various biological layers within living organisms. These layers encompass DNA, RNA, proteins, and metabolites, and their analysis is facilitated by high-throughput technologies (1–4). Recent advancements in both computational and experimental methodologies have substantially enhanced our capacity to profile multiple levels of cellular regulation, including the genome, transcriptome, epigenome, chromatin conformation, and metabolome, among other well-established “omics” (5–13). Consequently, these omics analyses have found widespread application in animal research in recent years (14, 15), as evidenced by the studies in cattle examining their role in production (16, 17), reproduction (18, 19), and metabolism/microbiome (20–22). Similarly, the contributions of omics techniques to understanding various traits in pigs (23–28), sheep, and goats (29) as well as chickens (30–32) have been comprehensively explored.

Furthermore, the utility of omics approaches extends to the authentication and assessment of various animal-derived products, including meat (33–40) and milk (41, 42). Surprisingly, despite these extensive investigations in multiple animal species, there is a notable absence of comprehensive reviews addressing the application of omics methodologies in donkey research. Therefore, this present review article aims to fill this gap by providing an overview of the genetic resources available for donkeys and examining the utilization of omics techniques in enhancing the productive and reproductive traits of donkeys in China. Additionally, this review delves into the role of omics applications in evaluating donkey-derived products, such as milk and meat.

## 2 Methodology for literature search

The central objective of this review article was to comprehensively investigate the evolving landscape of Omics applications within the domain of donkey research, with a particular emphasis on the Chinese context. This entailed conducting a systematic literature search to identify and assess pertinent studies, focusing exclusively on Chinese local donkey breeds. To ensure the selection of the most relevant literature, we devised stringent inclusion and exclusion criteria. The articles considered for inclusion were those published in recent years, which explored the utilization of Omics methodologies in livestock research, including pigs, cattle, sheep, goats, and poultry. Our review, however, honed in exclusively on studies related to donkeys, with a specific geographical focus on China. We meticulously chose a set of keywords that encapsulated the core themes of our review, including “donkey milk,” “donkey meat,” “donkey microbiome,” “donkey health,” “donkey reproduction,” and the overarching category of “omics.” Within the Omics umbrella, we included subdisciplines such as “genomics,” “transcriptomics,” “proteomics,” “metabolomics,” “microbiomics,” and “lipidomics.” Utilizing these keywords, we conducted an exhaustive search across reputable academic databases, including but not limited to PubMed, Scopus, Web of Science, and relevant academic journals. Following the initial search, the identified articles were subject to a rigorous screening process. Each article was assessed for relevance based on its title, abstract, and keywords. Only those articles meeting the criteria of investigating Omics applications in donkey research, particularly within the Chinese context, were retained. Relevant data from the selected articles were meticulously extracted and cataloged for subsequent analysis. This included information on the research objectives, methodologies employed, key findings, and any notable insights regarding the application of omics techniques in donkey research. The extracted data were synthesized, and patterns, trends, and advancements in the field of Omics applications in Chinese donkey research were critically evaluated. These insights were then organized and presented in a coherent manner within the review article.

## 3 Assessing the donkey welfare, distribution, and genetic resources in China

Donkey as a livestock animal has been ignored due to increased industrialization and mechanization because communities which previously relied on donkey traction now use motorized vehicles and machinery (43). In contrast, considerable attention has been given to the management and welfare and improvement of genetic resources and production of donkeys in China (44–49). According to our recent survey in different areas of China, we identified that there are well established and well-equipped donkey farms are existing in China (44). China realizes the importance of donkey as other livestock animals and still has reported 24 donkey breeds and number one in conservation of local donkey breeds [(48), https://zypc.nahs.org.cn/pzml/classify.html]. As per Food and Agricultural Organization report in 2020, the global donkey population was 50.45 million, of which 2.68 million are raised in China (47, 50). In China, donkeys are raised for milk, meat and hide production which might be another reason for the growing attention (44, 51–53).

The donkey is believed to have undergone a process of domestication approximately 5,000 years ago in Egypt, stemming from the African wild ass (54). This domestication event May have been prompted by environmental changes leading to a drier climate in the region. Previous research has suggested the possibility of dual domestication events, likely originating from the Nubian and Somali wild ass subspecies. These proposals are supported by patterns of mitochondrial DNA variation identified in both ancient and contemporary donkey populations (54–59). It is noteworthy that some extant subspecies of wild asses, as well as specific donkey breeds, face critical endangerment, leading to substantial conservation efforts (60). The pivotal contribution of genomics to the elucidation of the evolutionary history of equids has been comprehensively documented in a recently published studies (61–67). Consequently, researchers utilized the Chicago HiRise assembly technology to create a high-quality donkey genome assembly with sub-chromosomal scaffolds (60). This newly developed assembly has the potential to facilitate accurate assessments of heterozygosity in equine species beyond the horse, both at the genome-wide and local levels. Additionally, it aids in the detection of runs of homozygosity, which could be indicative of positive selection in domestic donkeys. Moreover, this advanced genome assembly enabled the identification of fine-scale chromosomal rearrangements between horses and donkeys, likely contributing to their divergence and eventual speciation (60). Recently, Liu et al. conducted genome-wide analyses using a novel donkey 40 K liquid SNP chip to study coat color diversity in the Chinese Dezhou donkey. However, SNP-Chip based for diversity purposes in donkey research is still in infancy (68).

China boasts a 4,000-year history of donkey husbandry and possesses abundant genetic resources in this domain (69–71). Animal genetic resources represent an indispensable facet of our genetic, economic, and cultural legacy, serving as a pivotal driver within the spheres of the economy, food production, regional identity, and ecosystem services (48, 72). Within this framework, it is crucial to recognize that local breeds, including those of donkeys, which May have lost their original purpose, are frequently confronting a dire existential threat. Consequently, there is an imperative need for concerted efforts aimed at their rehabilitation and reintegration into alternative economic utilization programs. Previous research has categorized Chinese donkey breeds into three regional branches: North China Plain, Loess Plateau, and Southwest China Plateau (73). The geographical distribution of these donkey breeds in China is primarily concentrated in Liaoning, Shanxi, Xinjiang, Inner Mongolia, Gansu, and Shandong Provinces (44, 48).

Recent research has identified a diverse array of donkey breeds within China’s genetic resources. These breeds encompass the Huaibei grey, Liangzhou, Qingyang, Changyuan, Biyang, Gunsha, Jiami, Guangzhong, Kulun, Xiji, Qinghai, Dezhou, Xinjiang, Hetian Grey, Turpan, Yangyuan, Taihang, Guangling, Jinnan, Linxian, Chuan, Xizang, Yunnan and Subei donkeys [(44, 74, 75), https://zypc.nahs.org.cn/pzml/classify.html]. These donkey breeds exhibit substantial variations in terms of body weight and size, with weight ranging from 130 to 260 kg and a height measuring between 110 cm to 130 cm. Morphologically, donkeys can be categorized into three main size groups: large-sized (Guanzhong, Hetian, Turpan, Changyuan, Jinnan, Guangling, and Dezhou donkeys), medium-sized (Jiami, Linxian, Biyang, Yangyuan and Qingyang donkeys), and small-sized (Kulun, Tibetan, Chuan, Subei, Huaibei, Xinjiang, Qinghai, Jiami Liangzhou, Taihang, Gunsha and Yunnan donkeys).^1^ These categories correspond to heights above 130 cm, between 115 cm and 125 cm, and below 110 cm, respectively (48). Among all donkey breeds, Dezhou donkey located in Shandong has extensively studied because of its heavy body, body height and length, predominantly black hair with a straight back and waist, arch of chest rib and round, firm hooves (44).

In China, traditional donkey breeding farms historically focused on a single local donkey breed. However, in a recent survey, 16 different local donkey breeds were identified. Notably, the Dezhou donkey currently dominates the Chinese donkey population, constituting over 57% of the total donkey population (44). This finding aligns with the findings of a prior study (48). In contrast, smaller-sized breeds such as Kulun, Qingyang, Xiji and Huaibei Grey donkeys constitute a significantly smaller proportion (44). This discrepancy is likely due to the relatively lower production value of meat and hide associated with these smaller breeds. Unfortunately, these smaller donkey breeds have faced indiscriminate slaughter in recent years, raising concerns about their potential extinction (57, 76). The details of donkey breeds and their location in China has been provided in Table 1. To preserve the genetic diversity of these smaller donkey breeds, it is crucial to establish dedicated breeding farms that are specifically designed for their conservation.

**Table 1:** Summary of donkey breeds and their location in China.

Breeds	Size [Height (cm)]	Location
Huaibei grey donkey	Male:116.12 ± 3.45	Anhui Province
	Female: 109.30 ± 4.89	
Liangzhou and Qingyang donkeys	Liangzhou donkey:	Gansu Province
	Male:108.90 ± 6.39	
	Female: 109.93 ± 8.63	
	Qingyang donkey:	
	Male:129.41 ± 2.52	
	Female: 124.93 ± 2.78	
Changyuan and Biyang donkeys	Changyuan donkey:	Henan Province
	Male:136.00 ± 3.40	
	Female: 129.40 ± 4.70	
	Biyang donkey:	
	Male:138.70 ± 5.40	
	Female: 131.40 ± 5.20	
Gunsha (Shanbei), Jiami and Guangzhong donkey	Gunsha donkey:	Shaanxi Province
	Male:115.65 ± 5.40	
	Female: 110.81 ± 5.69	
	Jiami donkey:	
	Male:126.80 ± 3.70	
	Female: 124.10 ± 3.70	
	Guangzhong donkey:	
	Male:133.45 ± 2.11	
	Female: 128.12 ± 4.82	
Kulun	Male:121.20 ± 1.93	Inner Mongolia
	Female: 110.12 ± 2.36	
Xiji donkey	Male:124.30 ± 4.60	Ningxia Province
	Female: 123.30 ± 6.10	
Qinghai donkey	Male:101.90 ± 9.34	Qinghai Province
	Female: 99.76 ± 7.51	
Dezhou donkey	Male:140.22 ± 3.80	Shandong Province
	Female: 135.03 ± 4.76	
Xinjiang, Hetian grey, and Turpan donkeys	Xinjiang donkey:	Xinjiang Province
	Male:181.30 ± 36.00	
	Female: 156.00 ± 31.10	
	Hetian grey donkey:	
	Male:132.00 ± 1.70	
	Female: 130.10 ± 3.33	
	Turpan donkey:	
	Male:141.20 ± 5.65	
	Female: 135.54 ± 4.82	
Yangyuan and Taihang Donkey	Yangyuan Donkey:	Hebei Province
	Male:133.60 ± 5.05	
	Female: 123.10 ± 8.44	
	Taihang Donkey:	
	Male:114.70 ± 8.64	
	Female: 104.22 ± 7.26	
Guangling, Jinnan and Linxian Donkeys	Guangling Donkey:	Shanxi province
	Male:141.40 ± 2.50	
	Female: 139.30 ± 3.80	
	Linxian Donkey:	
	Male:133.22 ± 3.73	
	Female: 133.16 ± 3.50	
	Linxian Donkey:	
	Male:124.00	
	Female: 123.60 ± 3.00	
Tibetan donkey	Male:102.86 ± 4.50	Tibet
	Female: 106.13 ± 8.50	
Chuan donkey	Male:98.73 ± 5.32	Sichuan province
	Female: 95.44 ± 4.28	
Yunnan donkey	Male:102.30 ± 5.72	Yunnan province
	Female: 98.89 ± 4.42	
Subei donkey	Male:122.60 ± 7.10	Jiangsu province
	Female: 118.40 ± 6.00	

The breeds and their location information were obtained from following published sources [(44, 48, 49, 58, 73–82), https://zypc.nahs.org.cn/pzml/classify.html].

## 4 Utilization of omics approaches in donkey milk production research

The research development on omics application in donkey milk research has been summarized in Table 2. New analytical technologies, with mass spectrometry being at the forefront, facilitate the generation of improved and innovative milk products based on the growing knowledge and understanding of milk bioactive compounds such as proteins, carbohydrates, lipids, and minerals, at global scale. The molecular understanding of biological milk function has emerged as a central theme in nutritional research (83–85). Mass spectrometry-based techniques enable the characterization of human and animal milk components not only in native fresh but also in processed milk. In recent years, the application of omics technologies has gained prominence in the field of donkey milk production research (86–92). Particularly, metabolomics, lipidomics, transcriptomics, and proteomics have played significant roles in advancing our understanding of various aspects of donkey milk and its potential applications. This paper provides a comprehensive overview of key findings and studies conducted in these areas.

**Table 2:** Summary of omics application in donkey milk research.

Omics techniques	Biological outcomes	Purpose	Reference
Metabolomics	Identification of metabolites and lipids in Chinese Liaoxi jennies colostrum and mature milk	Assessment of changes in milk composition during lactation	(100)
Metabolomics	Identification of differentially expressed metabolites in milk of two Dezhou donkey strains (SanFen and WuTou)	Exploration of difference in milk composition of two donkeys’ strains	(94)
Metabolomics	Metabolites profiling of bovine, goat and donkey milk extracellular vesicles	Composition and health benefit assessment of milk	(98)
Metabolomics	Screening of metabolites in donkey colostrum and mature milk	Evaluatation of changes in milk composition during lactation	(95)
Metabolomics	Screening of changes in free fatty acids profile in donkey colostrum and mature milk	For judgement of changes in milk composition during lactation	(97)
Metabolomics	Profiling metabolites in donkeys and human milk	Investigation of the suitability of donkey milk for human infant use	(93)
Proteomics	Differentially expressed proteins in donkey milk	Composition and functional exploration donkey milk	(148)
Proteomics	Differentially expressed amino acids in donkey colostrum and donkey mature milk associated with flavor and taste	The functionality and quality judgement of donkey milk	(149)
Proteomics	Screening whey proteins in donkey colostrum and mature milk	Evaluation of changes in milk composition with lactation	(107)
Proteomics	Milk fat globule membrane proteins in donkey milk	To examine the milk composition and its therapeutic efficacy assessment	(150)
LC–MS and GC–MS based Lipidomics	Lipids profiling of donkey milk in response to roughages feeding	Measurement of the changes in milk composition in response to roughages feeding	(151)

Metabolomic research in donkey milk has primarily focused on comparing and characterizing metabolite profiles. Significant studies have been conducted in the field of metabolomics, including notable investigations such as the examination of metabolite profiles in both donkey milk and human milk through GC–MS analysis (93). Additionally, Li et al. (94) conducted a metabolomic comparison involving two distinct Dezhou donkey strains, SanFen and WuTou, employing LC–MS methodology. Furthermore, an extensive analysis of metabolites within donkey milk throughout various stages of lactation was performed using un-targeted metabolomics coupled with ultra-high-performance liquid tandem chromatography quadrupole time-of-flight mass spectrometry (95). These studies shed light on the chemical composition of donkey milk, which is essential for assessing its nutritional value, bioactive compounds, technical properties, and potential diagnostic applications (96). Accordingly, a study employed a metabolomic approach to identify differentially free fatty acids and related signaling pathways in donkey milk across various lactational stages (97). Moreover, mass spectroscopy (MS) coupled with Ultrahigh-performance liquid chromatography (UHPLC) technique has been utilized to screen the metabolites in bovine, goat and donkey milk to assess the anti-inflammatory and immunoregulatory properties of milk extracellular vesicles (98). Consistently, a study found lipids and metabolites in milk and colostrum through metabolomics analysis by using UHPLC and MS (99).

Lipidomics, a vital field within metabolomics, aims to elucidate the structures of lipid molecules. Several studies have examined various lipid subgroups in donkey milk using liquid chromatography–tandem mass spectrometry, including fatty acids, polar lipids, and glycolipids (100, 101). Moreover, investigations into the differences in lipid composition of donkey milk at different lactation stages have been conducted (97, 99, 102). Notably, a study has compared the lipid profiles of donkey milk with those of cow and human milk (102). These studies have contributed valuable insights into the lipid contents of donkey milk.

The proteomics applications have been extensively discussed in donkey milk research (103–105). Furthermore, proteomic analysis has been employed to study differentially expressed whey proteins in donkey milk (106). These proteins have been linked to processes such as protein processing in the endoplasmic reticulum, estrogen signaling, progesterone-mediated oocyte maturation, and the PI3K-Akt signaling pathway (107). Studies have also reported differentially expressed whey proteins in donkey colostrum and mature milk, with implications for signaling and antigen processing pathways (95, 107). In a study utilizing the Equine 670 k Chip, a study successfully identified genes (NUMB, ADCY8, and CA8) associated with milk production traits in Xinjiang Donkeys (108). Consistently, proteomic analysis revealed some key differentially expressed proteins that were involved in regulation of complement and coagulation cascades, *staphylococcus aureus* infection and AGE-RAGE signaling pathways in diabetic complications (51). In addition, these proteins have key role in promoting cell proliferation, enhancing antioxidant, immunoregulation, anti-inflammatory, and antibacterial effects, and enhancing skin moisture (51). In addition a study emphasized the significance of proteomics and peptidomics in comparing proteins and endogenous peptides in human, cow, and donkey milk (109). Additionally, transcriptomic screening of donkey mammary glands has been used to identify molecular factors associated with reduced susceptibility to mastitis (110). Another study has investigated molecular mechanisms regulating bioactive milk components in mammary glands through transcriptomic profiling (111). In summary, the utilization of omics methodologies, encompassing metabolomics, lipidomics, transcriptomics, and proteomics, has made substantial contributions to our comprehension of donkey milk’s characteristics. These applications have enabled us to gain insights into the composition of donkey milk, its alterations in composition throughout the lactation period, and the ability to distinguish donkey milk from other sources. Furthermore, omics techniques have furnished us with invaluable insights into the constituent components and inherent qualities of donkey milk, thus establishing a solid groundwork for further scientific exploration and the potential for groundbreaking advancements in this particular field.

## 5 Utilization of omics approaches in donkey growth, meat production, and quality traits research

Recently a study conducted by Yu et al. (112) reported through transcriptomic screening several candidates Long non-coding RNAs (lncRNAs) that were involved in regulation of genes (DCN, ITM2A, MUSTN1, ARRDC2) associated with skeletal muscle development in donkeys (113). Consistently studies utilized genomic screening for polymorphisms and their genes that were associated with body size traits in Yangyuan donkeys (79) and chest circumference in Xinjiang Donkeys (114). In line with these studies, another study reported LCORL/NCAPG, FAM184B, TBX3, and IHH via Genomic screening which were associated with body height in Chinese 11 indigenous donkeys breeds (Biyang, Dezhou, Guangling, Hetian, Jiami, Kulun, Qingyang, Turpan, Tibetan, Xinjiang, and Yunnan) (115). While another study found eca-miR-1 regulated TMP3 gene via transcriptomic study, which is associated with skeletal muscle development (116). Besides, the omics methods have also been utilized to judge the quality of meat and changes in meat obtained from different sources of animals. The utilization of different omics techniques utilization in donkey growth and meat production and quality research have been summarized in Table 3.

**Table 3:** Summary of omics application in donkey meat production and growth traits research.

Omics techniques	Biological outcomes	Purpose	Reference
Genomics	Screening of SNPs associated with chest circumference	Improvement of meat production	(114)
Genomics	Identification polymorphisms and their genes associated with number of thoracic and lumber vertebrae	Enhancement of body size, carcass weight and meat quantity and quality	(152)
Genomics	Screening of genes associated body size traits	Acceleration the breeding potential of donkeys for meat production	(80, 115)
Transcriptomics	Identification differentially expressed Genes and miRNAs associated with Skeletal muscle development	Meat production improvement	(116)
Transcriptomics and Proteomics	Differentially expressed genes and proteins in longissimus dorsi muscles of donkey, cow, and goat	For assessing differences in meat of donkey, cow, and goat	(113)
UHPLC–ESI–MS and SPME–GC–MS based lipidomics	Lipid profiling of donkey, bovine, and sheep meat	Differentiating meat from different sources	(153)
Lipidomics	Lipid profiling of intramuscular fat in donkey	Assessing and enhancement of meat quality	(154)
Transcriptomics	Screening of genes associated with development of muscle fibre and tenderness	Improvement of meat quality	(155)
Proteomics	Screening of differentially expressed proteins in emitendinosus, longissimus thoracis and gluteus maximus muscles	Assessing and enhancement of meat quality	(156)
Transcriptomics	Screening of genes associated with skin thickness and muscle development	Improvement of Ejiao and meat production	(157)
Genomics	Screening of genes associated with body size	To enhance the adoptability and productive efficiency	(78)
Transcriptomics	Identification of miRNA regulated genes and their association with muscle fibre development	Enhancement of meat quality	(158)
Transcriptomics	Screening of genes associated intramuscular fat deposition	Improvement of meat quality	(81)
Proteomics	Proteins associated with intramuscular fat deposition	To enhance the quality of meat	(159)
Metabolomics and lipidomics	Screening of differentially expressed Lipids and metabolites in donkeys raw and cooked meat	For meat quality improvement	(160)
Transcriptomics and Lipidomics	Detection of differentially expressed genes and lipids in intramuscular fat and adipose tissue	Meat quality enhancement	(161)
LC–MS-Based Lipidomics	Lipid profiling of intramuscular fat in donkey	Assessing and enhancing the meat quality	(162)
Lipidomics	Screening of Differentially expressed Volatile compounds in various parts of Donkey meat and boiled meat	For assessing the quality of meat	(163)
lipidomics	Screening of Differentially expressed Volatile compounds and lipids in boiled meat of Donkey	For assessing the quality of meat	(164)
Transcriptomics	Identification of lysozyme gene in donkey breast and milk	Exploration of molecular mechanisms underlying phenotype traits.	(165)
Transcriptomics	Screening of candidate circular RNAs associated intramuscular fat contents development	Upgrading the meat quality	(166)
Transcriptomics	Screening of Long non-coding RNA (lncRNA) and their regulated genes associated with muscle development	Improvement of meat quality and quantity	(167)
Metabolomics	Changes in metabolites during the early postmortem aging in donkey meat	Exploring the quality of meat	(168)

## 6 Role of omics in donkey microbiota research

The gut microbiome of donkeys has garnered significant attention due to its pivotal role in donkey nutrition, as evidenced by recent studies (117, 118). A comparative investigation was conducted to assess the microbiota composition in Qinghai and Dezhou donkeys, revealing notable disparities. Specifically, it was observed that Qinghai Donkeys exhibited substantially higher flora diversity and richness in comparison to their Dezhou counterparts (119). While another study indicated that wild asses exhibit advantages over domestic donkeys in terms of dry matter digestion, gut microbial community composition, and function, it was also observed that wild asses possess a distinct intestinal flora adaptation for high altitudes on the Qinghai-Tibet plateau (120). This observation underscores the profound impact of gut microbiota on the adaptive evolution of donkeys. Utilizing advanced Omics techniques, multiple studies have consistently demonstrated a strong correlation between physiological variations and environmental changes with alterations in the gut microbiota of donkeys. These variations encompass diverse aspects such as pregnancy (121), age (122), transportation stress (122, 123) and altitude (118, 124, 125). Furthermore, these changes in gut microbiota composition and metabolite profiles have been shown to exert significant influence on maternal health, as well as the growth and development of the fetus (126–128). The relationship between feeding method and type of feed and the gut microbiota of weaned donkeys has been discussed in a recent study (129). Furthermore, the study by Huang et al. (130) found that supplementing donkeys with yeast polysaccharides significantly improved their gut microbiota and metabolites, which in turn were linked to enhanced immunity, better feed digestion, and improved growth in donkeys. Similarly, another study reported that high concentrate diet significantly improved the gut microbiota and metabolites following by enhanced of average daily gain and feed efficiency (131). By utilizing omics method, Zhang et al. (132) explored the differences in microbial diversity in small and large intestine and their impact on overall performance of donkeys (132). Recently our research team documented the dynamic changes in skin microbiota diversity and composition in donkeys of different ages and at different sites of the body (133). Collectively, these findings emphasize the imperative necessity of maintaining meticulous nutritional care and management practices for donkeys. Such measures are crucial not only for ensuring successful lactation but also for fostering optimal growth and health outcomes for both the donkeys and their foals. This scientific understanding underscores the significance of a comprehensive approach to donkey husbandry and underscores the importance of fostering a well-balanced gut microbiota for these animals’ overall well-being.

## 7 Omics application in donkey health research

Omics applications have gained prominence in the field of animal health (134), with recent attention being directed towards their utilization in donkey health research (135). Additionally, proteomic profiling in both neonatal and adult donkeys’ urine has proven valuable in assessing their health status (135). A multiomic approach has been employed to investigate the microbiota in the donkey hindgut, shedding light on its association with immunity and metabolism (117). Notably, whole genome sequencing has enabled the characterization of equine coronavirus obtained from donkeys with diarrhea in Shandong Province, China (136). In a study comparing jennies with and without reproductive issues, it was found that *Streptococcus zooepidemicus* isolates in jennies with reproductive problems exhibited a higher number of genes encoding virulence factors (137). Furthermore, a recent publication highlighted differentially expressed proteins in the serum of jennies with endometritis caused by *E. coli,* suggesting their potential as biomarkers for diagnosis (138). Despite these promising findings, it is evident that research in the realm of omics applications in donkey health remains limited, leaving ample room for further exploration and discovery in this domain.

## 8 Omics applications in donkey reproductive research

Genetic selection and breeding are crucial tools for livestock improvement (139). Consistently, the prospective utilization of genomics, with particular emphasis on its applicability to equine diseases and fertility, has been comprehensively documented (140). The transcriptomic screening of granulosa cells in response to heat stress has been reported in our previous study (141, 142). Consistently, a study reported differentially expressed genes in Donkeys granulosa cells in response to vitamins A, D and E and micronutrients (143, 144). Furthermore, they observed that most of the differentially expressed genes were associated with steroidogenesis and follicular development (143). A study utilized transcriptomic approach and documented differentially expressed genes in donkey oocyte that were majorly associated with RNA metabolism and apoptosis (145). These findings revealed the uniqueness of donkey conclude that, compared to other species, donkey oocytes express a large number of genes related to RNA metabolism to maintain normal oocyte development during the period from germinal vesicle to metaphase II. Consistently, a study through integrative screening of miRNA and mRNA and found several genes and microRNAs associated with spermatogenesis (146). Deoxynivalenol and zearalenone, which are commonly found in feed products, exhibit serious negative effects on the reproductive systems of domestic animals. A recent study utilized transcriptomic approach to explore their negative effect on donkey endometrial cells by down regulating the androgen and estrogen secretion-linked genes and upregulating the cancer-promoting genes (147). These genes could be utilized for improvement of donkey breeding in future. For ease of reviewing, we have summarized the research development on omics application in donkey reproduction in Table 4.

**Table 4:** Summary of omics applications in donkey reproduction.

Omics techniques	Biological outcomes	Purpose	Reference
Transcriptomics	Identification of genes associated with oocyte development	Physiology of oocyte across various animal species	(145)
Transcriptomics	Screening of genes associated with male reproductive cells development and sperm physiology	Genetic markers for molecular breeding	(146)
Transcriptomics	Screening of genes regulated in response to deoxynivalenol and zearalenone and their effect on reproductive cells function	Genetic response of reproductive cells to deoxynivalenol and zearalenone	(147)
Transcriptomics	Identification of genes and lncRNAs associated with testicular development and spermatogenesis	Improvement of donkey production via molecular breeding	(169)
Transcriptomics	Profiling of genes and miRNAs associated with development of testicular tissue and male reproductive traits	Genetic markers identification for enhancement of donkey breeding efficiency	(170)
Transcriptomics	Identification of Circular RNAs (circRNAs) and their genes associated with spermatogenesis and testes development	Genetic markers for molecular breeding	(171)
Transcriptomics	Screening of differentially genes associated with development and maturation of oocytes	Improvement of donkey production through molecular breeding	(172)
Proteomics	Seminal plasma proteins are essential for sperm function and also related to individual differences in sperm freezability. Tandem Mass Tag (TMT) proteomics screening of differentially expressed proteins profile was reported in response to various freezing methods in seminal plasma	Investigate the molecular mechanisms of donkey sperm cryotolerance	(173)
Proteomics	Profiling of differentially expressed proteins in fresh and frozen–thawed spermatozoa	The sperm viability enhancement and prevention the possible injuries exploration during cryopreservation	(174)
Proteomics	Differentially expressed proteins profile was reported in response to various freezing methods in seminal plasma	The evaluation of molecular mechanisms of donkey sperm cryotolerance	(175)
Metabolomics	Profiling of differentially expressed metabolites in fresh and frozen–thawed spermatozoa	To enhance the viability of sperm and prevent the possible injuries during cryopreservation	(176)

## 9 Conclusion

Various omics technologies such as genomics, transcriptomics, metabolomics, lipidomics, and proteomics, have been used in different areas of donkey research. These include genetic resources, milk production, growth, meat quality, microbiota, health, and reproduction. However, it is important to note that the application of omics methods in donkey research is still in its early stages. There is significant potential for further exploration and discoveries in this field. Therefore, future research should concentrate on harnessing the potential of omics technologies to improve donkey health, productivity, and genetic conservation.

## Footnotes

^1^ https://zypc.nahs.org.cn/pzml/classify.html
